# IL-24 modulates the high mobility group (HMG) A1/miR222 /AKT signaling in lung cancer cells

**DOI:** 10.18632/oncotarget.11838

**Published:** 2016-09-02

**Authors:** Janani Panneerselvam, Akhil Srivastava, Ranganayaki Muralidharan, Qi Wang, Wei Zheng, Lichao Zhao, Alshine Chen, Yan D. Zhao, Anupama Munshi, Rajagopal Ramesh

**Affiliations:** ^1^ Department of Pathology, The University of Oklahoma Health Sciences Center, Oklahoma City, Oklahoma 73104, USA; ^2^ Department of Radiation Oncology, The University of Oklahoma Health Sciences Center, Oklahoma City, Oklahoma 73104, USA; ^3^ Department of Biostatistics and Epidemiology, The University of Oklahoma Health Sciences Center, Oklahoma City, Oklahoma 73104, USA; ^4^ Stephenson Cancer Center, The University of Oklahoma Health Sciences Center, Oklahoma City, Oklahoma 73104, USA; ^5^ Graduate Program in Biomedical Sciences, The University of Oklahoma Health Sciences Center, Oklahoma City, Oklahoma 73104, USA

**Keywords:** IL-24, lung cancer, HMGA1, miR222-3p, metastasis

## Abstract

Interleukin (IL)-24, a novel tumor suppressor/cytokine exhibits antitumor activity against a broad-spectrum of human cancer cells. In a recent study, we showed that IL-24 inhibited AKT in lung cancer cells. However, the molecular mechanism of AKT inhibition by IL-24 remains elusive.

The high mobility group (HMG) A1 a member of the non-histone chromosomal proteins and commonly referred to as architectural transcription factor, regulates transcription of various genes involved in cell growth and survival. Overexpression of HMGA1 has been shown to be associated with tumor progression and metastasis in several cancers, including human lung cancer. A recent study demonstrated that HMGA1 activates AKT function by reducing the activity of the protein phosphatase, phosphatase 2A subunit B (PPP2R2A) *via* the oncogenic micro (mi) RNA-222. Based on this report we hypothesized that IL-24-mediated AKT inhibition involved the HMGA1/miR-222 axis.

To test our hypothesis, in the present study we used a H1299 lung cancer cell line that expressed exogenous human IL-24 when induced with doxycycline (DOX). Induction of IL-24 expression in the tumor cells markedly reduced HMGA1 mRNA and protein levels. Using a mechanistic approach, we found that IL-24 reduced miR-222-3p and -5p levels, as determined by qRT-PCR. Associated with HMGA1 and miR-222 inhibition was a marked increase in PPP2R2A, with a concomitant decrease in phosphorylated AKT^T308/S473^ expression. SiRNA-mediated knockdown of HMGA1 in combination with IL-24 significantly reduced AKT ^T308/S473^ protein expression and greatly reduced cell migration and invasion compared with individual treatments. Further combination of IL-24 and a miR-222-3p inhibitor significantly increased PPP2R2A expression.

Our results demonstrate for the first time that IL-24 inhibits AKT *via* regulating the HMGA1/miR-222 signaling node in human lung cancer cells and acts as an effective tumor suppressor. Thus, a therapy combining IL-24 with HMGA1 siRNA or miR-222-3p inhibitor should present effective treatment of lung cancer.

## INTRODUCTION

Lung cancer remains the major cause of cancer-related deaths in men and women worldwide. Although considerable advancements have been made in systemic treatments [1–5] and targeted therapy for translocations and mutations [6], the overall clinical outcome remains dismal [7, 8]. One major reason for poor prognosis is tumor invasion and metastasis [9]. Therefore, to control lung cancer metastasis effectively, it is crucial to identify novel target molecules involved in cancer cell growth and survival, thereby improving the clinical outcomes of metastatic lung cancer.

High mobility group (HMG) A1 proteins are architectural nuclear factors, and are the key players in the assembly of transcriptional factors and cofactors to form enhanceosomes [10, 11]. HMGA proteins bind to the minor groove of AT-rich DNA sequences through AT hooks in the N-terminal region of the protein. Thereby it alters the chromatin structure and regulates the transcriptional activity of several genes [10, 11]. Growing evidence has illustrated the association between HMGA1 overexpression and cancer metastasis in a range of human cancers, such as colorectal cancer [12], ovarian cancer [13], head and neck tumor [14], glioblastomas [15], pancreatic cancer [16], breast cancer [17], prostate cancer [18], and lung cancer [19]. However, the expression of HMGA1 is minimal or absent in normal adult tissues [20]. This differential expression of HMGA1 in neoplastic and normal cells allows for the specificity and low toxicity of HMGA1-based treatment strategies. *In vitro* and *in vivo* studies have shown that inhibiting HMGA1 expression with antisense oligonucleotide reduced cancer cell invasion/migration and increased apoptotic cell death [21–23]. Further, HMGA1 silencing promoted cancer cell chemo sensitivity [24, 25]. Therefore, targeting HMGA1 could be an excellent strategy to inhibit lung tumor cell survival and metastasis.

Studies have demonstrated that HMGA1 overexpression activates AKT and its associated function in cancer cells [21, 26, 27]. AKT is a key downstream effector of HMGA1-dependent signaling and provides critical cell survival signals for tumor progression by phosphorylating several proteins involved in cell cycle regulation and pro-apoptotic factors [21, 26–28]. A recent report revealed mechanistic evidence of HMGA1-activated AKT function by reducing the activity of the protein phosphatase PPP2R2A *via* the oncogenic micro (mi) RNA-222 [28]. Further, it has been shown that pharmacologic and biological inhibition of AKT/mTOR signaling suppressed cancer cell migration, invasion, and metastasis [29–31].

The human melanoma differentiation-associated gene (mda)-7/IL-24 is a unique cytokine/tumor suppressor gene that belongs to the IL-10 cytokine family [32]. IL-24 expression is lost in most cancer cells of human origin [32]. Studies have shown that loss of IL-24 expression correlated with disease progression in melanoma and lung cancer, indicating a tumor suppressive role for IL-24 [33, 34]. *In vitro* and *in vivo* studies in a broad spectrum of human cancer cells demonstrated that exogenous IL-24 expression has anti-tumor, anti-angiogenic, and anti-metastatic properties and suppresses various signaling pathways, without harming normal cells [35–37]. Further, the efficacy of IL-24 as an anti-cancer drug was demonstrated in a Phase I clinical trial using an adenovirus-mda-7 (INGN-241)-based cancer gene therapy approach [38].

In the present study, we examined the effect of IL-24 on HMGA1 expression. Our recent observation of IL-24-mediated AKT inhibition in lung cancer cells [37] and results from another study indicating that the HMGA1/miR-222 axis is involved in AKT regulation prompted this line of investigation [28]. We hypothesized that IL-24 inhibits AKT by regulating the HMGA1/miR-222 axis in non-small cell lung cancer (NSCLC). Moreover, we theorized that IL-24 would exhibit enhanced anti-metastatic activity when combined with HMGA1 siRNA and miR-222-3p inhibitor.

## RESULTS

### HMGA1 and IL-24 expression in primary lung tumors and in cultured human lung cancer cells

**Figure 1:**
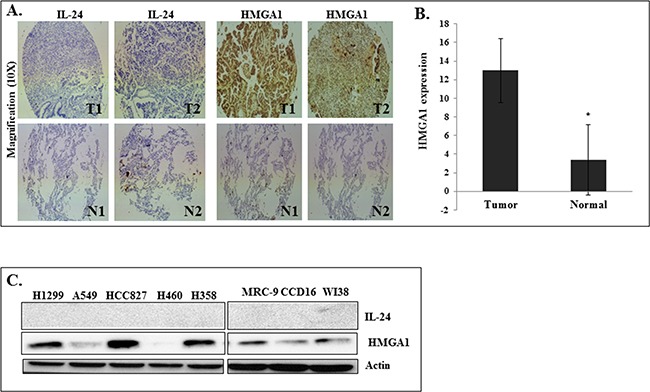
HMGA1 and IL-24 expression in human primary lung tumors and in cultured human lung cancer cells **A.** Immunohistochemical staining for IL-24 and high mobility group AT-hook 1 (HMGA1) in human lung cancer and normal lung tissues. T-Tumor tissue, N-Normal tissue. **B.** Quantitative analysis of HMGA1 expression in lung tumor and normal tissues. Asterisk denotes significance (*p*<0.0001). **C.** Expression of IL-24 and HMGA1 proteins in human lung cancer and normal lung cell lines.

To assess IL-24 and HMGA1 protein expression in normal lung and lung tumor tissues, we performed immunohistochemistry (IHC) in a commercially available tissue microarray (TMA; BC041115b; US Biomax, Inc.), consisting of paired samples of lung cancer tissues and corresponding normal tissues. We observed that IL-24 was not detectable in all lung cancer tissues, with slight expression in normal lung tissues. In contrast, strong nuclear and higher HMGA1 expression was observed in lung cancer tissues compared to the expression in normal lung tissues (Figure 1A, 1B). While we could show HMGA1 and IL-24 expression in the TMAs, we could not correlate the expression with clinical outcome, mutation status or smoking history as they were not available from the company that supplied the TMA. Nevertheless it is evident that HMGA1 expression was higher in tumor tissues compared to normal while IL-24 was not detectable in tumor tissues independent of histology. Further, we observed a strong and inverse correlation between HMGA1 gene expression and overall survival in patients diagnosed with lung cancer. Kaplan-Meier survival curve analysis of 1926 lung cancer patients showed that patients with high HMGA1 gene expression had low overall survival compared to patients with low HMGA1 expression (Supplementary Figure S1) [39].

Further, the expression levels of IL-24, and HMGA1 proteins were analyzed in cultured human lung cancer cells (H1299, A549, HCC827, H460, and H358) and human normal lung fibroblasts (MRC-9, CCD16, and WI38). Expression levels of HMGA1 varied among the cancer cell lines (Figure 1C). H1299, HCC827, and H358 displayed increased HMGA1 expression, compared with normal lung cell lines. However, all lung cancer cell lines tested was negative for endogenous IL-24 expression, which is consistent with our TMA staining results from human lung tumors (Figure 1A, 1B).

### IL-24 decreases HMGA1 expression in H1299-IL24^wt^ cells

**Figure 2:**
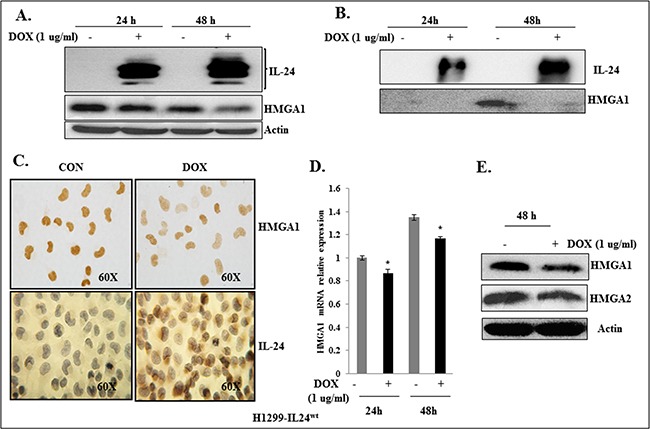
IL-24^wt^ reduced HMGA1 expression in H1299-IL24^wt^ lung cancer cells **A.** IL-24^wt^ reduced HMGA1 expression at 24 h and 48 h in DOX-treated H1299-IL24^wt^ cells, but not in untreated control cells. **B.** Secreted HMGA1 expression is inhibited by IL-24^wt^ in conditioned medium at 24 h and 48 h in DOX-treated H1299-IL24 cells, but not in untreated control cells. **C.** Immunocytochemistry showed that DOX-induced IL-24^wt^ expression in H1299-IL24^wt^ cells reduced HMGA1 expression. **D.** RT-PCR analysis showed that IL-24^wt^ reduced HMGA1 mRNA levels at 24 h and 48 h. **E.** IL-24^wt^ reduced expression of HMGA2, in DOX-treated H1299-IL24^wt^ cells compared with expression of these proteins in untreated H1299-IL24^wt^ cells. Asterisk denotes significance (*p*<0.01).

To determine the inhibitory activity of IL-24 on HMGA1, we used the H1299 cell line (labeled “H1299-IL24^wt^”), which was stably transfected with doxycycline (DOX)-inducible plasmid vector expressing wild-type IL-24 (pTET-IL-24^wt^) as described previously [40]. H1299-IL24^wt^ cells were treated with 1 μg/ml DOX to express IL-24^wt^ at 24 h and 48 h. The expression of IL-24^wt^ resulted in a marked reduction in HMGA1 expression at both time points (Figure 2A). Since HMGA1 is a secreted protein, we also analyzed the inhibitory effect of IL-24^wt^ on HMGA1 expression in the conditioned medium, and observed a decrease at 48 h (Figure 2B). Immunocytochemical staining demonstrated that the induced expression of IL-24^wt^ reduced HMGA1 expression (Figure 2C) in H1299-IL24^wt^ cells compared with control cells that were not induced to express IL-24^wt^. This observation concurs with our western blot data.

Next to determine the effect of IL-24^wt^ on HMGA1 mRNA level, we used RT-PCR analysis to assess HMGA1 mRNA expression in H1299-IL24^wt^ cells. Induction of IL-24^wt^ significantly reduced HMGA1 mRNA expression at 24 h and 48 h compared with controls (*p*<0.01;Figure 2D). Together, our data strongly suggest that IL-24^wt^ regulates HMGA1 at mRNA and protein levels.

Since HMGA1 and HMGA2 are splice variants and have overlapping functions [10, 11], we also analyzed the inhibitory effect of IL-24^wt^ on HMGA2 expression. We found IL-24^wt^ in addition to reducing HMGA1 also reduced HMGA2 expression (Figure 2E).

### IL-24 attenuates HMGA1 and its downstream target AKT in lung cancer cells but not in normal cells

Studies have shown that HMGA1 primarily mediates cancer progression through PI3K-AKT signaling [21, 27, 28], which plays a significant role in tumor growth, survival, metastasis, and therapy resistance [29–31, 41]. Most importantly, AKT has been reported as the main downstream target of HMGA1 and has been shown to be positively regulated by HMGA1 overexpression in cancer cells [21, 26–28]. Therefore, we tested whether suppression of HMGA1 by IL-24^wt^ altered AKT expression.

**Figure 3:**
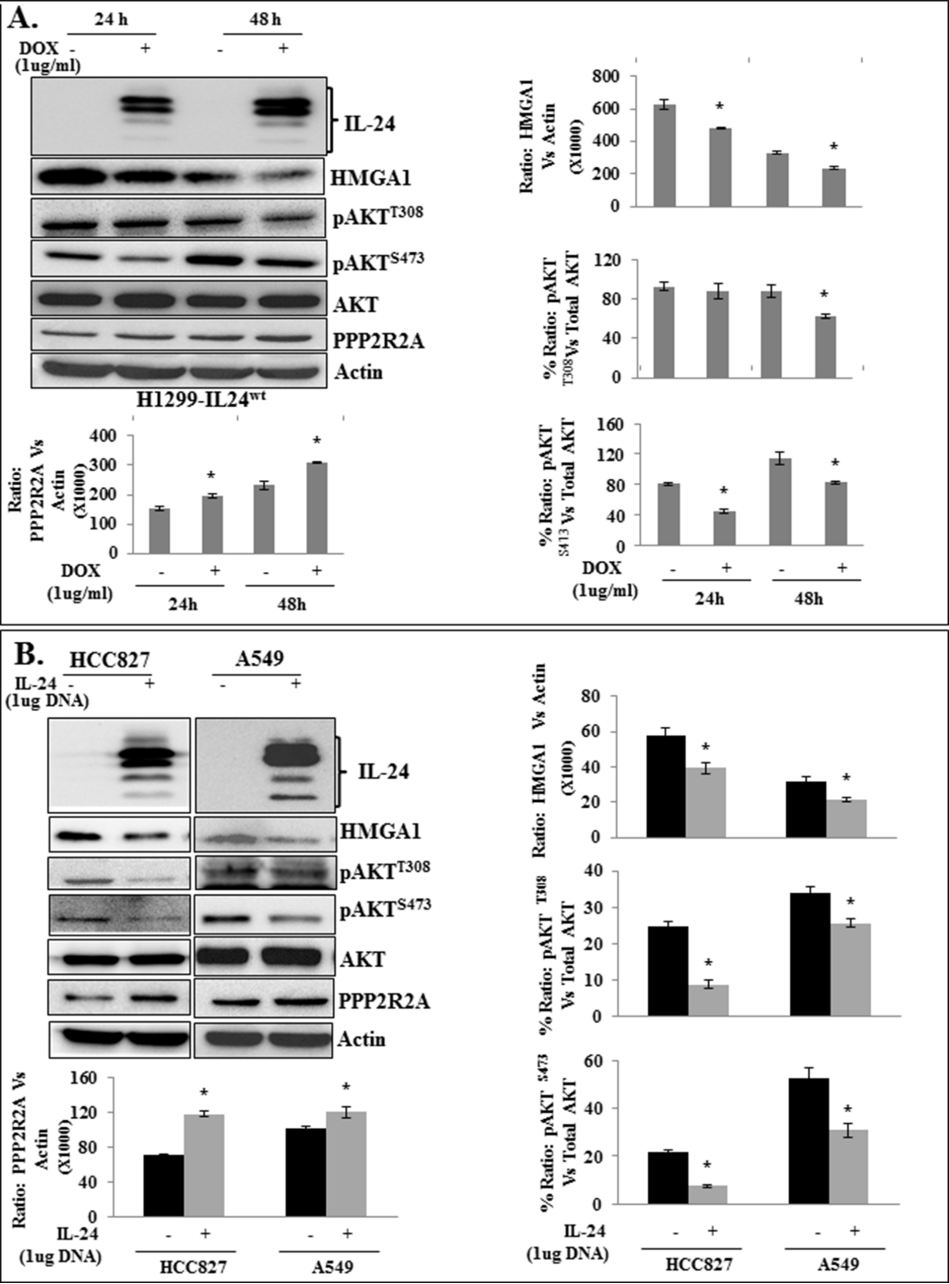
Attenuation of HMGA1 by IL-24^wt^ inhibits AKT activation in H1299-IL24 lung cancer cells **A.** Western blotting analysis showed that expression of IL-24^wt^ protein in H1299-IL24 cells reduced the expression of phosphorylated (p) AKT^T308^ and pAKT^S473^, and increased PPP2R2A expression at 24 h and 48 h after doxycycline treatment. **B.** Western blotting analysis showed that expression of IL-24^wt^ protein in HCC827 and A549 cells reduced the expression of phosphorylated (p) AKT^T308^ and pAKT^S473^, and increased PPP2R2A expression. Beta-actin was used as a protein loading control. Differences in the expression of the proteins were determined by semi-quantitative analysis and represented in graphical format (*p*<0.05). Bars denote standard deviation (*SD*).

A decrease of HMGA1 expression by IL-24^wt^ in H1299-IL24^wt^ cells resulted in a marked reduction in the expression levels of pAKT^T308^ at 48 h and pAKT^S473^ at 24 h and 48 h compared with control cells (*p*<0.01;Figure 3A). Furthermore, with IL-24^wt^ induction was a concomitant increase in protein phosphatase PPP2R2A expression (Figure 3A). These observations demonstrate that IL-24^wt^-mediated HMGA1 inhibition attenuates AKT while enhancing PPP2R2A expression. Thus IL-24^wt^ inhibits HMGA1 and its downstream signaling molecules that are involved in cancer progression

To determine whether the observed IL-24^wt^-mediated attenuation of HMGA1 and its axis was unique to H1299 cells, we performed experiments in additional lung cancer cell lines, HCC827 (p53-mutated) and A549 (p53-wild type). HCC827, A549, and H1299 cells were transiently transfected with an IL-24^wt^-expressing plasmid DNA vector. The cells were harvested and cell lysates were analyzed by western blotting. Untransfected cells served as controls. Compared with control cells, all three transfected cell lines expressed IL-24^wt^ (Figure 3B, Supplementary Figure S2). A marked reduction in HMGA1 protein expression was associated with IL-24 expression in all three cell lines (*p*<0.01). In addition, a reduction in pAKT^T308^ and pAKT^S473^ expression was observed in IL-24^wt^-expressing cells, with a concomitant increase in PPP2R2A expression compared with controls (*p*<0.01; Figure 3B, Supplementary Figure S2). These results show that suppression of HMGA1 by IL-24^wt^ is not unique to one cell line, and that IL-24-mediated inhibitory activity on HMGA1 can be attained by both inducible and transient expression of IL-24^wt^.

Next, to determine whether the observed inhibitory effect on HMGA1 axis occurs solely by IL-24^wt^, we conducted experiments using the H1299-IL24^mt^ cell line. This cell line is stably transfected with DOX-inducible plasmid vector expressing mutant (mt) IL-24 (pTET-IL-24^mt^), in which all five phosphorylation sites of IL-24 are mutated, as described previously [40]. In H1299-IL24^mt^ cells, IL-24^mt^ protein expression produced no significant change in HMGA1, pAKT^T308^, and pAKT^S473^ expression at 24 h, but revealed a marked increase at 48 h after DOX treatment (Supplementary Figure S3). This finding suggests that wild-type IL-24, but not mutant IL-24, is involved in the regulation of the HMGA1 axis.

In order to exclude the possibility that DOX treatment alone could directly inhibit HMGA1, we treated naïve H1299 cells with 1 μg/ml doxycycline. DOX treatment did not inhibit HMGA1 expression, compared with controls (Supplementary Figure S4).

We also tested the impact of exogenous expression of IL-24^wt^ on HMGA1 and its downstream targets in normal human lung fibroblasts (MRC-9). We observed no significant difference in protein expression levels of control and IL-24^wt^-expressing cells (Supplementary Figure S5). These results demonstrated that IL-24^wt^ selectively regulated the HMGA1/AKT axis in tumor cells but not in normal cells.

### IL-24^wt^ combined with siRNA-mediated HMGA1 inhibition exhibited greater inhibition of HMGA1 signaling in cancer cells

**Figure 4:**
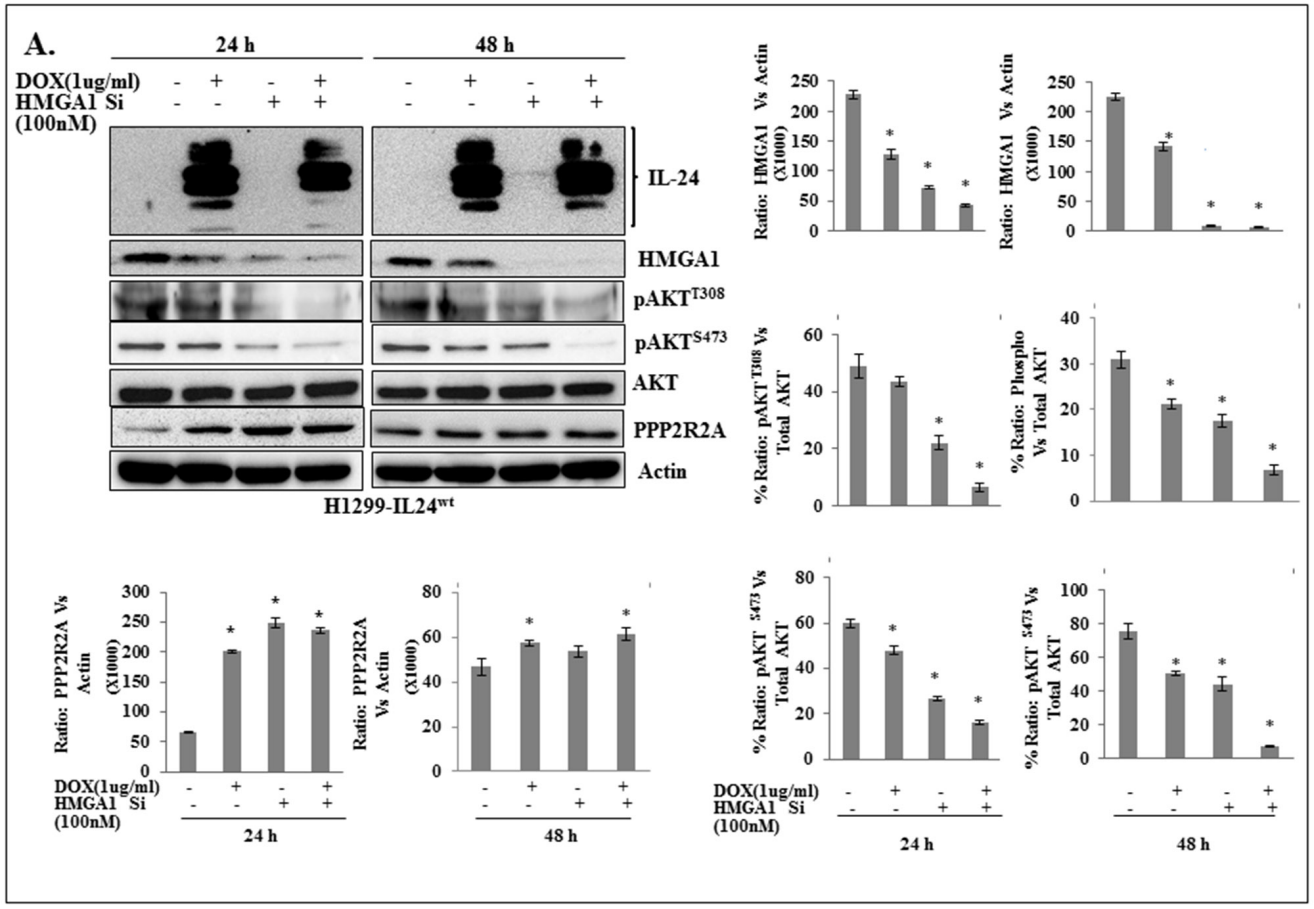

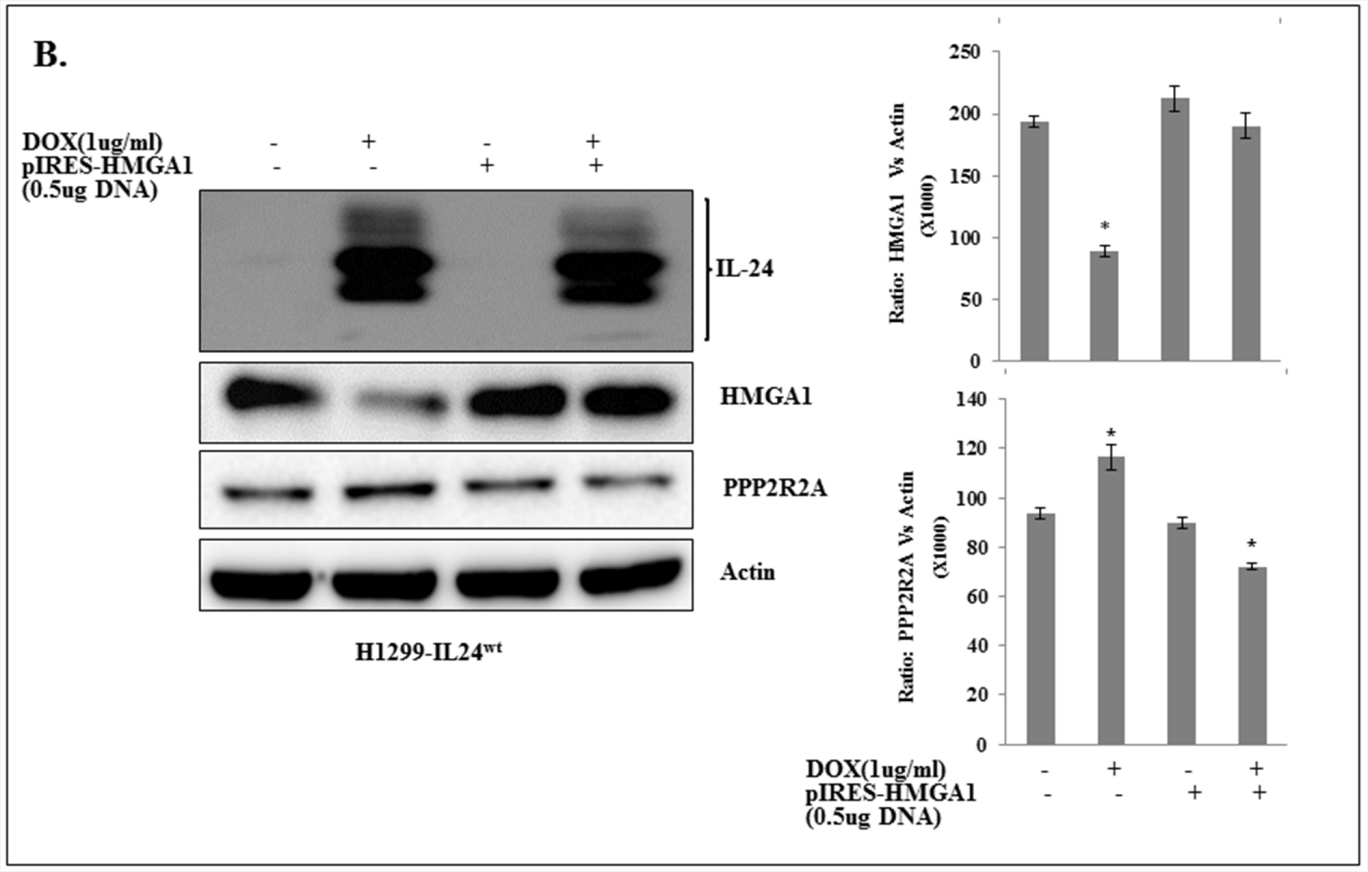
A combination of IL-24^wt^ and HMGA1-siRNA showed greater inhibition of AKT activation in lung cancer cells **A.** Western blotting showed that a combination of HMGA1 siRNA and IL-24^wt^ produced a greater reduction in HMGA1, pAKT^T308^ and pAKT^S473^ expression compared with all other treatments. Beta-actin was used as a protein loading control. Differences in the expression of the proteins against untreated control were determined by semi-quantitative analysis and represented in graphical format (*p*<0.001). Bars denote standard deviation (*SD*). **B.** H1299- IL-24^wt^ cells were untransfected or transfected with pIRES-HMGA1 plasmid, followed by treatment with or without 1 μg/ml doxycycline. Cells were subjected to western blot analysis. Expression of pIRES-HMGA1 plasmid abrogated the inhibitory effect of IL-24^wt^ on HMGA1 and the associated effect on its downstream target PPP2R2A. Beta-actin was used as a protein loading control. Differences in the expression of the proteins were determined by semi-quantitative analysis and represented in graphical format (*p*<0.05). Bars denote standard deviation (*SD*).

Since IL-24 attenuated the HMGA1 signaling axis, we next investigated the combined inhibitory effect of IL-24^wt^ and genetic knockdown of HMGA1 by utilizing siRNA on the HMGA1 axis as a treatment strategy in lung tumor cells. HMGA1, pAKT^T308^, and pAKT^S473^ protein expression were reduced, with an associated increase in PPP2R2A expression, in the cells induced with IL-24^wt^ alone, HMGA1 siRNA alone, and IL-24^wt^ plus HMGA1 siRNA, compared with control cells (*p*<0.01; Figure 4A). However, greater inhibition of HMGA1, pAKT^T308^, and pAKT^S473^ protein expression was observed in cells that were treated with IL-24^wt^ plus HMGA1 siRNA than in cells that received individual treatment (*p*<0.01; Figure 4A).

To determine whether the observed combined inhibitory effect of IL-24 and HMGA1 siRNA on HMGA1 signaling was restricted to one cell line, we performed experiments in an additional HCC827 lung cancer cell line. H1299 and HCC827 cells were transiently transfected with HMGA1 siRNA, with or without IL-24^wt^-expressing plasmid DNA vector. Cell lysates were collected and were analyzed using western blotting. Untransfected cells served as controls. In both H1299 and HCC827 cells, the transfection of IL-24^wt^ plasmid DNA vector and HMGA1 siRNA inhibited HMGA1 signaling similar to that observed in H1299-IL24^wt^ cells as evidenced by reduced HMGA1, pAKT^T308^, and pAKT^S473^ protein expression (Supplementary Figure S6 and S7). These results indicate that the combined enhanced inhibitory effect of IL-24^wt^ and genetic knockdown of HMGA1 is not unique to one cell line. Further, the results indicate the potential for testing combinatorial therapy for lung cancer treatment.

We also tested whether IL-24^wt^ had an inhibitory effect when HMGA1 is overexpressed. HMGA1 overexpression was achieved by transfecting H1299-IL-24^wt^ cells with pIRES-HMGA1 plasmid. We observed a significant increase in HMGA1 expression with a decrease in PPP2R2A expression in pIRES-HMGA1 plasmid-overexpressing cells, compared with vector controls (Figure 4B). However, we did not observe any IL-24-mediated change in HMGA1 or PPP2R2A expression in HMGA1 plasmid-overexpressing cells. These results suggest the existence of a threshold for IL-24^wt^-mediated inhibitory activity on HMGA1 over and above which it fails to have a suppressive effect on HMGA1. This observation further supports the combinatorial therapy might be more effective in controlling HMGA1 and its downstream signaling for having an effective therapeutic outcome.

### IL-24^wt^ in combination with HMGA1 siRNA treatment exhibited enhanced inhibition of tumor cell migration and invasion

**Figure 5:**
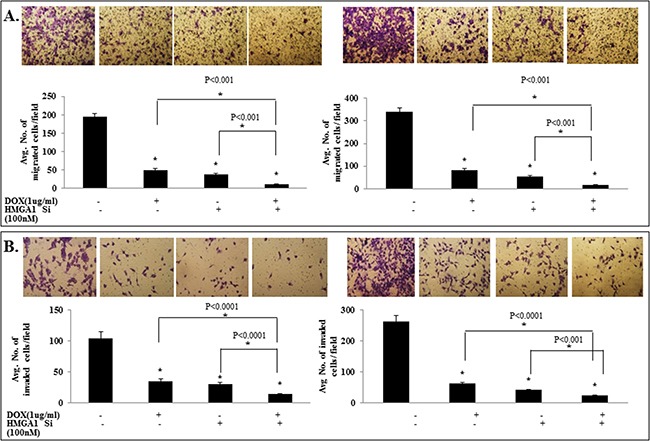
IL-24^wt^ in combination with HMGA1 siRNA treatment exhibited enhanced inhibition of tumor cell migration and invasion si-RNA-mediated HMGA1 knockdown combined with IL-24^wt^ resulted in significant reduction of tumor **A.** cell migration and **B.** invasion compared with controls. IL-24 treatment alone and HMGA1 siRNA treatment alone significantly inhibited tumor cell migration and invasion compared with controls (*p*<0.0001).

We have previously shown IL-24^wt^ reduced tumor cell migration and invasion [33]. Further, studies have shown that both HMGA1 and AKT play an important role in lung cancer metastasis [13, 22]. Therefore, we tested the inhibitory effects of IL-24^wt^ and HMGA1 siRNA treatment on cell migration and invasion. As shown in Figure 5, the combination treatment of IL-24 and HMGA1 siRNA showed the highest inhibition of tumor cell migration (*p*<0.001; Figure 5A) and invasion (*p*<0.001; Figure 5B), compared with all other treatments. These results demonstrate that attenuation of HMGA1 by combining IL-24^wt^ and siRNA is more effective in inhibiting the HMGA1 axis and its mediated cell migration and invasion than either approach alone.

Next, we determined whether overexpressing HMGA1 will rescue the IL-24^wt^-mediated inhibitory effects on cell invasion. HMGA1 overexpression resulted in a significant increase in cell invasion compared to untreated controls while IL-24^wt^-inhibited cell invasion (*p<*0.05; Supplementary Figure S8). However, HMGA1 overexpression completely and significantly abrogated the IL-24^wt^-mediated inhibition of cell invasion (*p<*0.05; Supplementary Figure S8). These results show that the IL-24^wt^ inhibitory activity on the HMGA1 axis has a threshold over and above which its suppressive activity is abrogated.

### Modulation of miR-222-3p enhances IL-24-mediated inhibition of HMGA1 signaling in H1299-IL24^wt^ cells

**Figure 6:**
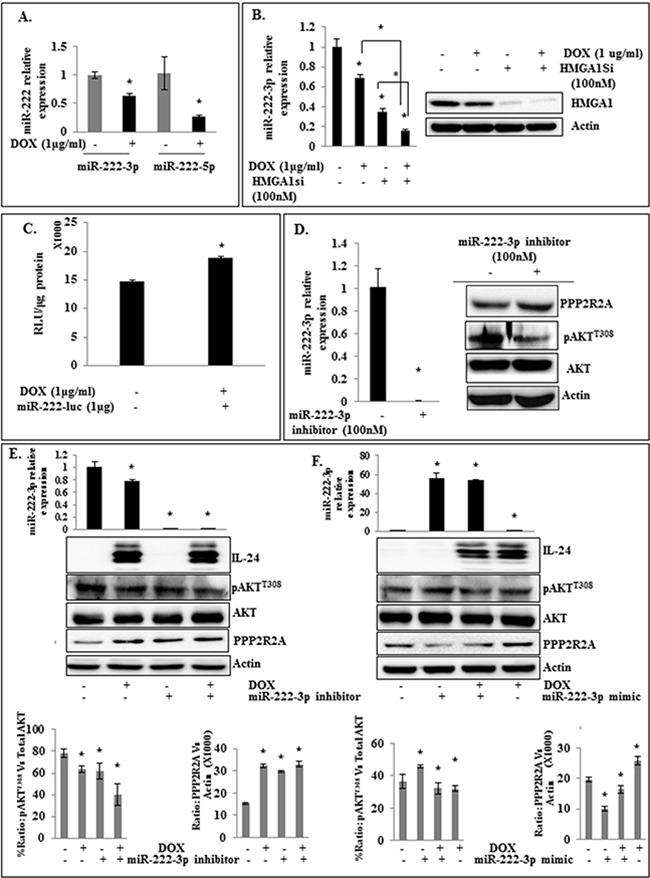
Modulation of miR-222-3p enhances the inhibitory activity of IL-24^wt^ on HMGA1 signaling in H1299-IL24^wt^ cells **A.** Induction of IL-24^wt^ downregulates miR-222-3p and -5p expression in H1299-IL-24^wt^ cells (*p*<0.05). **B.** IL-24^wt^ shows greater inhibition of miR-222-3p expression when combined with genetic knockdown of HMGA1 (*p*<0.05). **C.** The expression of IL-24^wt^ in the presence of miR-222-3p-Luc plasmid produced an increase in luciferase activity compared with the miR-222-3p-Luc plasmid alone group (*p*<0.0001). **D.** Treatment of H1299-IL-24^wt^ cells with miR-222-3p inhibitor increased PPP2R2A protein expression. **E.** IL-24^wt^ combined with miR-222-3p inhibitor exhibited a greater increase in PPP2R2A expression (*p*<0.05). **F.** IL-24 expression did not reduce miR-222-3p expression in cells overexpressing miR-222-3p mimic (*p*<0.05). Further, we observed increased PPP2R2A expression after IL-24^wt^ treatment in miR-222-3p-overexpressing cells when compared with miR-222-3p mimic alone group (*p*<0.0001). Beta-actin was used as a protein loading control.

HMGA1 overexpression has recently been shown to correlate with the increased expression of oncogenic miR-222 [28]. HMGA1 enhances its expression by directly binding with the proximal promoter of miR-222 [28]. Further, siRNA-mediated HMGA1 silencing significantly downregulated miR-222 expression. In addition, miR-222 overexpression has been shown to downregulate PPP2R2A in non-small lung cancer cells by directly binding to the 3'-UTR region, thereby activating AKT signaling [28]. Since IL-24 inhibited HMGA1 expression and its downstream target AKT, we next raised the question of whether IL-24-mediated AKT inhibition involves inhibition of miR-222 expression. H1299-IL-24^wt^ cells induced with DOX showed a significant reduction in the expression of both miR-222-3p (predominant form) and miR-222-5p (less abundant form; *p*<0.05; Figure 6A). This showed that IL-24^wt^-mediated HMGA1 inhibition repressed its direct downstream target miR-222 in H1299-IL-24^wt^ cells. Further, IL-24^wt^ when combined with HMGA1 siRNA produced the highest inhibition of miR-222-3p expression when compared to individual treatments (*p*<0.001; Figure 6B).

Next, we used a luciferase-based reporter assay to confirm that IL-24^wt^ represses miR-222-3p expression. We used miRNA luciferase reporter, which has binding sites for miR-222-3p at 3'UTR downstream of the reporter luciferase gene. When miRNA is expressed, it binds to 3'UTR, resulting in the repression of luciferase gene expression. The combination of IL-24^wt^ expression with pMiR-222-3p-Luc plasmid increased luciferase activity, compared with pMiR-222-3p-Luc plasmid alone (*p*<0.0001; Figure 6C). This finding indicates the IL-24^wt^ mediated the reduced expression of miR-222-3p in lung tumor cells, and is consistent with our RT-PCR data (Figure 6B).

Next, we treated H1299-IL24^wt^ cells with miR-222-3p inhibitor. We observed a significant reduction in miR-222-3p expression that was accompanied with a concomitant increase in PPP2R2A expression (*p*<0.0001; Figure 6D). Supporting our findings, other studies have shown that miR-222 repression upregulated PPP2R2A and its associated downregulation of p-AKT [28]. Together, these data suggest that the observed IL-24^wt^-mediated AKT inhibition occurs through suppression of the HMGA1/miR-222-3p node.

Since IL-24^wt^ attenuated HMGA1/miR-222-3p axis signaling, we next investigated the effect of a combination of IL-24^wt^ and miR-222-3p inhibitor on AKT signaling in H1299-IL-24^wt^ cells. We demonstrated a significant decrease in miR-222-3p expression in cells transfected with miR-222-3p inhibitor compared with controls (*p*<0.05; Figure 6E). We also observed a further reduction in miR-222-3p expression when IL-24^wt^ was added to miR-222-3p inhibitor-treated cells. A decrease in pAKT^T308^ and increase in PPP2R2A protein expression was also observed in the cells treated with IL-24^wt^ plus miR-222-3p inhibitor compared with controls and those treated with either agent alone (*p*<0.05; Figure 6E). The possibility of non-specific inhibitory activity of miR-222-3p inhibitor was eliminated by using a control inhibitor (negative control) that did not show any activity on miR-222-3p or on AKT expression compared to miR-222-3p inhibitor (Supplementary Figure S9A).

Finally, we examined whether IL-24^wt^ could inhibit AKT when miR-222-3p is overexpressed using miR-222-3p mimic. Prior to conducting the studies, we first tested the specificity of miR-222-3p mimic by transfecting H1299-IL24^wt^ cells and comparing with a control mimic. Increased miR-222-3p and pAKT^T308^ expressing was observed in miR-222-3p mimic-treated cells compared to control mimic-treated and untreated cells (Supplementary Figure S9B). Based on the results we next examined the inhibitory effects of IL-24^wt^ in miR-222-3p mimic treated H1299-IL24^wt^ cells. The overexpression of miR-222-3p was confirmed by RT-PCR analysis. We observed a marked increase in pAKT^T308^ and decrease in PPP2R2A expression in miR-222-3p-overexpressing cells compared with controls (Figure 6F). IL-24^wt^ treatment alone produced a marked decrease in pAKT^T308^ and increase in PPP2R2A expression accompanied with reduced miR-222-3p expression (*p*<0.05; Figure 6F). However, IL-24^wt^ did not produce a significant reduction in miR-222-3p expression nor increased the expression of PPP2R2A in cells expressing miR-222-3p mimic when compared to IL-24^wt^ treatment alone. A slight increase in PPP2R2A expression was observed in the combination treatment group when compared to miR-222-3p mimic treated cells without IL-24^wt^ but was still lesser than the untreated control (Figure 6F). Our results demonstrate that IL-24^wt^ is more effective in inhibiting the HMGA1/miR-222-3p/AKT axis when it is combined with miR-222-3p inhibitor and inefficient to override when miR-222-3p mimic is expressed.

## DISCUSSION

Lung cancer-related death is primarily due to drug-resistance and metastasis [7, 8]. Current treatments are ineffective and is evident from the poor five-year survival rate of lung cancer patients [9]. Therefore, there is an urgent need to identify new therapeutic targets that represent a molecular determinant of cellular invasiveness and approaches to suppress those targets. Studies have shown that overexpression of HMGA1 proteins is associated with lung cancer metastasis through transcriptional upregulation of genes involved in the promotion of metastasis in various tumor types [12–19]. Liau et al. [15] showed that HMGA1 primarily mediates its cellular invasiveness through the PI3K/AKT/mTOR signaling pathway, which is involved in cancer cell proliferation, survival, chemo-resistance, and metastasis, and that specific suppression of HMGA1 inhibits cellular invasiveness *in vitro* and metastasis *in vivo* through AKT inhibition [21–23]. AKT is a serine-threonine kinase; upon phosphorylation at T308 and S473, AKT activates several target proteins that are involved in tumor cell proliferation, apoptosis, and the cell cycle [41, 42]. Deregulation of the PI3K/AKT/mTOR pathway is highly involved in lung carcinogenesis, as this pathway is extensively interconnected with other oncogenic signaling [41, 43–45]. Therefore, HMGA1, a regulator of the PI3K/AKT/mTOR pathway, is a potential target for lung cancer treatment.

IL-24 is a novel tumor suppressor gene that has demonstrated anti-tumor effects in a broad spectrum of human cancers [40–42]. Recently, we reported that IL-24 effectively suppressed AKT/mTOR signaling and its associated tumor cell migration [39]. However, the mechanism by which IL-24 mediated AKT inhibition is poorly understood. We thus speculated that IL-24 might inhibit AKT activation through HMGA1 suppression. In the present study, we determined whether (i) IL-24 mediates inhibition of HMGA1 expression in lung cancer cells and (ii) IL-24 mediates AKT inhibition through HMGA1 suppression. We found that IL-24^wt^ mediated significant downregulation of HMGA1 protein expression. In addition, our results showed that the IL-24^wt^-mediated inhibition of HMGA1 protein expression was not unique to one cell line. Comparative analysis between stably transfected and transiently transfected IL-24 in cancer cells revealed similar effects on HMGA1 expression. This finding confirms that IL-24 expression is critically involved in regulating HMGA1 expression in lung cancer cells. Further, RT-PCR analysis showed that IL-24 reduced HMGA1 at the mRNA level. This observation indicated that IL-24 modulates HMGA1 protein expression by reducing the level of HMGA1 mRNA.

Studies have reported that siRNA-mediated HMGA1 silencing produced a significant upregulation of PPP2R2A expression, with associated downregulation of pAKT [28]. However, HMGA1 overexpression promoted constitutive AKT activation [28]. These observations are consistent with our results showing that HMGA1 regulates AKT activation. Studies using si-HMGA1 and pIRES-HMGA1 plasmid overexpression demonstrated that IL-24 inhibits AKT by attenuating HMGA1 expression. Further, HMGA1 inhibition produced an increase in PPP2R2A expression. PPP2R2A protein is a phosphatase that inactivates AKT function by inducing AKT dephosphorylation [28]. This could be the reason for the inhibition of AKT upon HMGA1 attenuation. Further, the combination of IL-24 and HMGA1 silencing more strongly inhibited AKT signaling and the associated tumor cell migration and invasion.

In the present study although we have shown IL-24 inhibited AKT by suppressing HMGA1, it is possible that other signaling proteins such as CXCR4 that are known to regulate AKT are also modulated by IL-24. We recently reported IL-24 downregulates the SDF-1/CXCR4 axis and its downstream AKT signaling and thereby inhibiting lung tumor cell migration and invasion [37]. Thus, it is evident from the previous report [37], and the present study that AKT is the common downstream target for both SDF/CXCR4 and HMGA1 pathway and that IL-24 inhibits AKT activation by attenuating CXCR4 and HMGA1 expression. What remains unknown and is of interest is to determine whether there is cross-talk between HMGA1 and CXCR4 and can HMGA1 directly regulate CXCR4 or *vice versa*. Information available in this area will aid not only in understanding how cancer cells may escape HMGA1 or CXCR4 targeted therapies but will also provide a rationale for combinatorial therapies targeting both HMGA1 and CXCR4. A report indicates that silencing HMGA1 represses expression of CXCR4 indicating occurrence of cross-talk [46]. However, details of this study are not available and hence will need to be explored further.

Zhang et al. [28] have demonstrated that HMGA1 regulates PPP2R2A expression, either directly by repressing PPP2R2A transcription, or indirectly through enhancing miR-222 expression. They revealed that overexpression of HMGA1 was significantly associated with increased levels of miR-222 in lung tumor specimens and cell lines [28]. Further, they showed that HMGA1 silencing reduced miR-222 transcriptional activity, whereas forced HMGA1 expression increased miR-222 expression by directly binding with the proximal promoter of miR-222 in NSCLC cell. MiR-222 has been reported to inhibit PPP2R2A protein expression in lung tumor cells by directly binding to its 3'UTR region [28].

MicroRNAs (miRNAs) are small noncoding RNAs that play a critical role in basic biological and pathological processes [47, 48]. MiRNAs regulate the expression of target genes at the post-transcriptional level through degradation of transcripts and inhibition of translation, mainly by binding to 3'-UTR of target messenger RNA (mRNA) [47, 48]. Accumulating evidence has demonstrated that miR-222 is overexpressed in several types of cancers, including lung cancer [28, 49–53]. In non-small cell lung cancer, miR-222 expression is reported to be associated with cancer cell proliferation, metastasis, drug resistance, and poor survival [28, 54]. In addition, miR-222 is reported to be involved in the activation of various pathways, including AKT signaling, in cancer cells [55]. Consistent with these findings, our results demonstrated that miR-222-3p expression decreased as a consequence of IL-24^wt^-mediated HMGA1 expression, and was associated with upregulation of PPP2R2A expression. Studies using miR-222-3p inhibitor and mimic showed that IL-24^wt^ mediates AKT inhibition in lung cancer cells by regulating the HMGA1/miR-222 node. To our knowledge, this is the first report to highlight that IL-24^wt^ attenuates AKT through suppression of the HMGA1/miR-222 axis in lung cancer cells. Further, we have shown that the observed effects are specific to wild-type IL-24 as the mutant IL-24 protein did not demonstrate similar inhibitory activity on HMGA1, AKT or miR-222.

Further, studies have shown that HMGA1 overexpression is involved in the dysregulation of numerous oncogenic genes and miRNAs in many tumor types, including lung cancer [56]. With this in mind, combination therapy may be more effective for the treatment of metastatic lung cancer than the individual therapeutic agents. Supporting this, our results showed marked inhibition of HMGA1/miR-222/AKT signaling upon treatment with a combination of IL-24^wt^ with either HMGA1 siRNA or miR-222-3p inhibitor.

In conclusion, our results showed that IL-24^wt^ inactivates AKT by suppressing the HMGA1/miR-222 axis and that the combination of IL-24^wt^ with HMGA1 silencing and miR-222 inhibitors was more effective in attenuating AKT and the associated lung cancer migration and invasion. Finally, our study results provide a basis for the development of an IL-24/HMGA1-based therapeutic approach for lung cancer treatment.

## MATERIALS AND METHODS

### Cell culture

Human non-small cell lung cancer cell (NSCLC) lines (H1299, A549, HCC827, H460, and H358) and normal lung fibroblast cells (MRC-9, CCD16, and WI38) (American Type Culture Collection (ATCC), Manassas, VA, USA) were cultured as previously described [36, 37, 40]. The cell lines were authenticated at the Genetic Resource Core Resource Facility, Johns Hopkins University, Baltimore, MD. In all experiments, untreated cells served as controls.

### Stable transfection of inducible IL-24 plasmid vector in H1299 cells

Creation of IL24^wt^ and IL24^mt^-inducible plasmids and H1299-IL24^wt^ and H1299-IL24^mt^ cell lines were previously described [40]. These plasmids and cell lines were used in our studies.

### Transient transfection of IL-24 plasmid

NSCLC cells (H1299, A549, and HCC827), and normal lung fibroblast cells (MRC-9) were seeded in six-well tissue culture plates and were transiently transfected with 1 μg of plasmid expression vector carrying the IL-24 cDNA using DOTAP:cholesterol liposomes, as previously described [37]. After six hours of transfection, tissue culture medium was aspirated and replenished with fresh medium. Untransfected cells served as controls. The cells were harvested at 24 h and 48 h after transfection. Cell lysates were prepared and used for protein expression analysis.

### Transient transfection of oligonucleotides

H1299-IL24^wt^ (1 X 10^5^) cells were seeded in six-well plates and were transiently transfected with the miR-222-3p inhibitor and miR negative control (100 nM; Dharmacon) using DOTAP:Cholesterol liposome as previously described [37, 40]. After six hours of transfection, tissue culture medium was aspirated and replenished with fresh medium. The cells were then either not treated or treated with doxycycline (1 μg/ml). Untransfected cells served as controls. The cells were harvested at 24 h after transfection. Cell lysates were prepared and used for miRNA and protein expression analysis.

H1299-IL24^wt^ (1 X 10^5^) cells were seeded in six-well plates and were either treated or not treated with doxycycline (1 μg/ml, Sigma Chemicals). After six hours, the medium was removed and cells were transiently transfected with miR-222-3p mimic and miR negative control (50 nM, Dharmacon) using DOTAP:Cholesterol liposome as previously described [37, 40]. Cells that were not transfected served as controls. The cells were harvested 24 h after transfection. Cell lysates were prepared and used for miRNA and protein expression analysis. Negative controls used in our miR222-3p overexpression/downregulation studies, are chemically synthesized single stranded modified RNAs. These negative controls have no homology to any known mammalian gene. Use of non-targeting inhibitors and mimics in miRNA inhibition and mimic experiments respectively will distinguish between specific inhibitor/mimic activity and their background effects. Neg.con. - denotes Negative control.

### HMGA1 RNA interference studies

H1299-IL24^wt^ (1 X 10^5^) cells were seeded in six-well plates and transfected with 100 nm of HMGA1siRNA (Dharmacon) using DOTAP:Cholesterol liposome as previously described [37, 40]. Six hours after transfection, the medium was replaced with RPMI-1640 containing 2% tetracycline-free serum, with or without 1 μg/ml of doxycycline. Untransfected cells served as controls. After 24 h of incubation, the cells were harvested and total cell lysates were prepared for miRNA and protein expression analysis.

HCC827 and H1299 cells were seeded in six-well plates and transfected with 50 and 100 nM of HMGA1 siRNA, respectively, with or without 1 μg of plasmid expression vector carrying the IL-24 cDNA using DOTAP:Cholesterol liposome, as previously described [37, 40]. Six hours after transfection, the tissue culture medium was aspirated and replenished with fresh medium. Untransfected cells served as controls. After 24 h of incubation, the cells were harvested and total cell lysates were prepared for protein expression analysis.

### HMGA1 overexpression studies

H1299-IL24^wt^ (1 X 10^5^) cells were seeded in six-well plates and transfected with 0.5 μg pIRES-HMGA1 DNA (Addgene) using DOTAP:Cholesterol liposome, as previously described [37, 40]. Six hours after transfection, the medium was replaced with RPMI-1640 containing 2% tetracycline-free serum, with or without 1 μg/ml of doxycycline. Untransfected cells served as controls. After 48 h of incubation, the cells were harvested and total cell lysates were prepared for protein expression analysis.

### Cell migration and invasion assay

A cell migration assay utilizing polycarbonate filters with a pore size of 8 μm (BD Biosciences, Bedford, MA, USA) and an invasion assay using matrigel pre-coated filters with a pore size of 8 μm (BD Biosciences) were performed as previously described [37, 40]. Briefly, H1299-IL24^wt^ cells (5×10^4^) were seeded in the upper chambers of the migration and invasion inserts and were transfected with HMGA1 siRNA (100 nM). After six hours of transfection, cells were either untreated or treated with doxycycline (1 μg/ml). The lower chamber was filled with 20% tetracycline-free FBS-containing medium. After 24 h and 48 h of incubation, the numbers of migrated and invaded cells were counted, as previously described [37, 40].

For determining whether the IL-24-mediated inhibitory activity could be rescued by overexpressing HMGA1, H1299-IL24^wt^ cells (5×10^4^) were seeded in the upper chamber of the invasion inserts and were either transfected with pIRES-HMGA1 plasmid DNA (0.5 μg) or not transfected. After six hours of transfection, the cells were either untreated or treated with doxycycline (1 μg/ml). Cells that were not transfected and not treated with doxycycline served as controls. The lower chamber was filled with 20% tetracycline-free FBS-containing medium. After 48 h of incubation, the numbers of invaded cells were counted, and subjected to statistical analysis.

### Immunocytochemistry (IHC)

H1299-IL24^wt^cells (1 x 10^4^) were seeded on Lab-Tek 2-well chamber slides (Nalge-Nunc International, Rochester, NY, USA) and were either untreated (control) or treated with doxycycline (1 μg/ml), and were stained as previously described [36, 37]. Primary antibodies used were mouse anti-human IL-24 antibody (1:1000) (Introgen Therapeutics, Houston, TX, USA) and rabbit anti-human HMGA1 antibody (1:2000) (Cell Signaling Technology Inc; Beverly, MA, USA). The slides were cover-slipped and examined. Photographs were captured using a Nikon TiU microscope (Nikon Instruments Inc., Melville, NY, USA).

### Tissue microarray and IHC analysis

A lung cancer tissue microarray (US Biomax, BC041115b) containing 41 cases of lung squamous cell carcinoma, 2 specimens of lung adenosquamous carcinoma, 48 specimens of lung adenocarcinoma, 4 specimens each of lung bronchioalveolar carcinoma and large cell carcinoma, 6 samples of small cell undifferentiated carcinoma, 5 carcinoid specimens, and 10 normal lung tissue samples was stained with mouse anti-human IL-24 antibody (1:1000) and rabbit anti-human HMGA1 antibody (1:2000), following a previously described immunohistochemistry protocol [36, 37]. The slides were cover-slipped and examined. Images were captured using a Nikon TiU microscope (Nikon Instruments Inc., Melville, NY, USA). Each stained tissue micro-section was reviewed by a pathologist, and blindly scored based on the percent of stained tissue (1 for <10%; 2 for < 25%; 3 for <50% - 4 for > 60% positive staining). The individual score for each tissue section was then summed and represented as the average score for tumor and normal tissues.

### Survival curve analysis

The correlation between overall survival (OS) and HMGA1 expression was examined by Kaplan-Meier (K-M) survival plot using freely available Kaplan–Meier plotter (www.kmplot.com).

### Luciferase reporter assay

H1299-IL24^wt^cells (1 x 10^5^) were seeded in six-well tissue culture plates, were transiently transfected with 1 μg of pMiR-222-3p-Luc plasmid (Signosis, Inc., Santa Clara, CA, USA), and were encapsulated in cationic DOTAP:Cholesterol liposome [37, 40]. After six hours of transfection, the tissue culture medium was removed and replenished with fresh medium supplemented with or without doxycycline (1 μg/ml). At 48 h after doxycycline treatment, the medium was removed and cells were washed gently with PBS. Cells were scraped and supernatants were collected. Into 96-well white (opaque) plates, cell lysates from each sample were transferred and 100 μl of luciferase assay reagent was added. Luciferase activity was measured with a Perkin Elmer EnVision Multilabel Reader (Waltham, MA, USA), according to the manufacturer's instructions. For each sample, the results from triplicate wells were calculated and are presented as the average of triplicate samples. Experiments were performed independently a minimum of three times in order to determine statistical significance.

### Real-time PCR analysis

Total RNA from the control and doxycycline-treated H1299-IL24^wt^ cells was isolated using Trizol (Life Technologies, Grand Island, NY, USA) and was subjected to reverse transcription using an iScript cDNA synthesis kit (Bio-Rad, Hercules, CA, USA). The complementary DNA (cDNA) was subsequently used to perform real-time (RT)-PCR (Bio-Rad CFX96 Touch Real-Time PCR Detection System) with SYBR chemistry using iQTM SYBR Green super mix (Bio-Rad) and human HMGA1-specific oligonucleotide primers (Forward-5'GCCTGGGATCTGAGTACATATTG 3'-Sense, Reverse-CGGAAGCAAAGTAGGGTTAGG3'-AntiSense; Integrated DNA Technologies, Coralville, IA, USA). Thermal cycling was programmed as follows: 95°C for 30 s, followed by 40 cycles of 95°C for 20 s, 62°C for 20 s, and 72°C for 20 s. The crossing threshold (Ct) value assessed by RT-PCR was noted for the transcripts and normalized with human 18S mRNA (Forward- 5'-CAGCCACCCGAGATTGAGCA-3' and Reverse- 5'-TAGTAGGGACGGGCGGTGTG-3'; Integrated DNA Technologies). The changes in mRNA were expressed as fold changes relative to control ± the standard deviation (*SD*).

For quantitative analysis of miRNA, total miRNA isolated from H1299-IL24^wt^ and H1299-IL24^mt^ cells were subjected to reverse transcription with Superscript™ II RNase H - Reverse transcriptase and universal primers (Qiagen). Complementary DNA (cDNA) was subsequently used to perform RT-PCR with SYBR™ chemistry (Qiagen) using specific primers for miR-222-3p and 5p. The crossing threshold value assessed using RT-PCR was noted for the transcripts and was normalized with SNORD95 miRNA. The changes in miRNAs were expressed as fold changes relative to the control value ±*SD*.

### Western blotting analysis

Cells receiving various treatments and collected at various time points were subjected to western blot analysis as previously described [37, 40]. Primary antibodies against IL-24 (1:2000; Introgen Therapeutics, Houston, TX, USA), HMGA1 (Cat. No. 7777), HMGA2 (Cat. No. 8179), phospho-AKT^S473^(Cat. No. 4060), total AKT (Cat. No. 9272) and PPP2R2A (Cat. No. 5689) were all purchased from Cell Signaling Technology Inc.; Beverly, MA, USA; phospho-AKT^T308^ (Cat. No. 38449; Abcam) and STAT-3 (Santa Cruz), and beta actin (1:2000; Sigma Chemicals) were purchased and used as recommended by the manufacturers. Proteins were detected using the appropriate secondary antibodies (Santa Cruz Biotechnology, Inc., and Jackson Immuno-Research Laboratories, Inc., West Grove, PA, USA) and an enhanced chemiluminescence kit (Thermo Scientific). Protein levels were detected using a chemiluminescence imaging system (Syngene, Frederick, MD) and quantified using gel quant software.

### Statistical analysis

Unless otherwise stated, all data are shown as mean ± standard deviation (*SD*). Univariate statistical significance was determined by one-way analysis of variance (ANOVA) with Tukey's adjustment for pairwise comparisons. Differences between groups were obtained using a linear mixed effects model with Tukey's adjustment. All quantitative data are expressed as mean ± *SD* of a minimum of three independent experiments. A p-value of less than 0.05 was considered statistically significant. SAS 9.2 was used for the statistical analyses.

## SUPPLEMENTARY FIGURES


