# Supplementary material for: IL-24 modulates the high mobility group (HMG) A1/miR222 /AKT signaling in lung cancer cells
Source: Oncotarget. 2016 Sep 2;7(43):70247–63. doi: 10.18632/oncotarget.11838 (PMC5342550; doi:10.18632/oncotarget.11838)
Supplement: Supplementary file 1 [file oncotarget-07-70247-s001.pdf]

IL-24 modulates the high mobility group (HMG) A1/miR222 / AKT signaling in lung cancer cells

SUPPLEMENTARY FIGURES

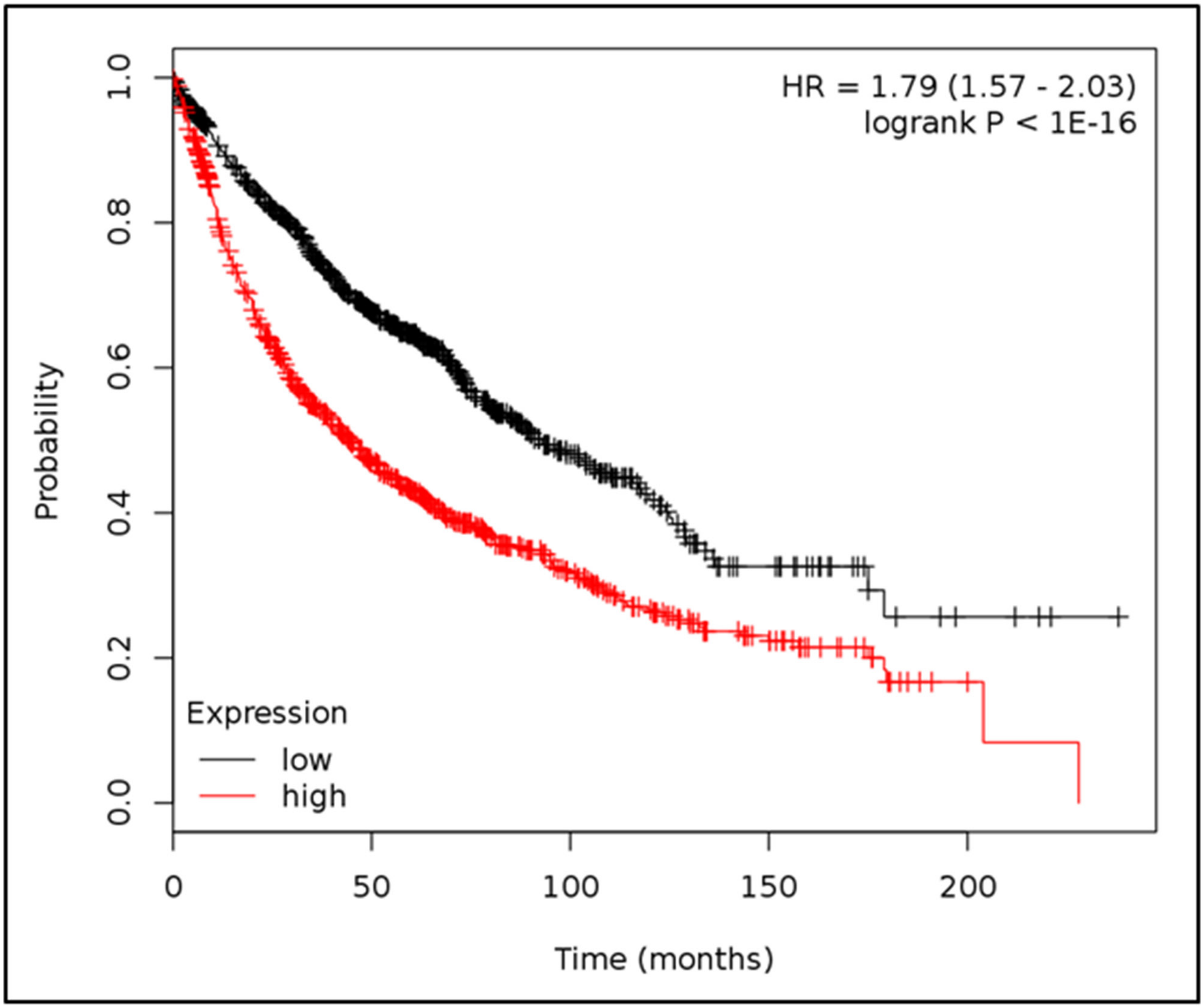

Supplementary Figure S1: HMGA1 expression correlates with overall survival of lung cancer patients. Correlation between HMGA1 gene expression and overall survival was determined in 1926 lung cancer patients using the K-M plot. Kaplan-Meier survival curve show patients with low HMGA1 expression had a higher overall survival than those with high expression (logrank p<1E-16).

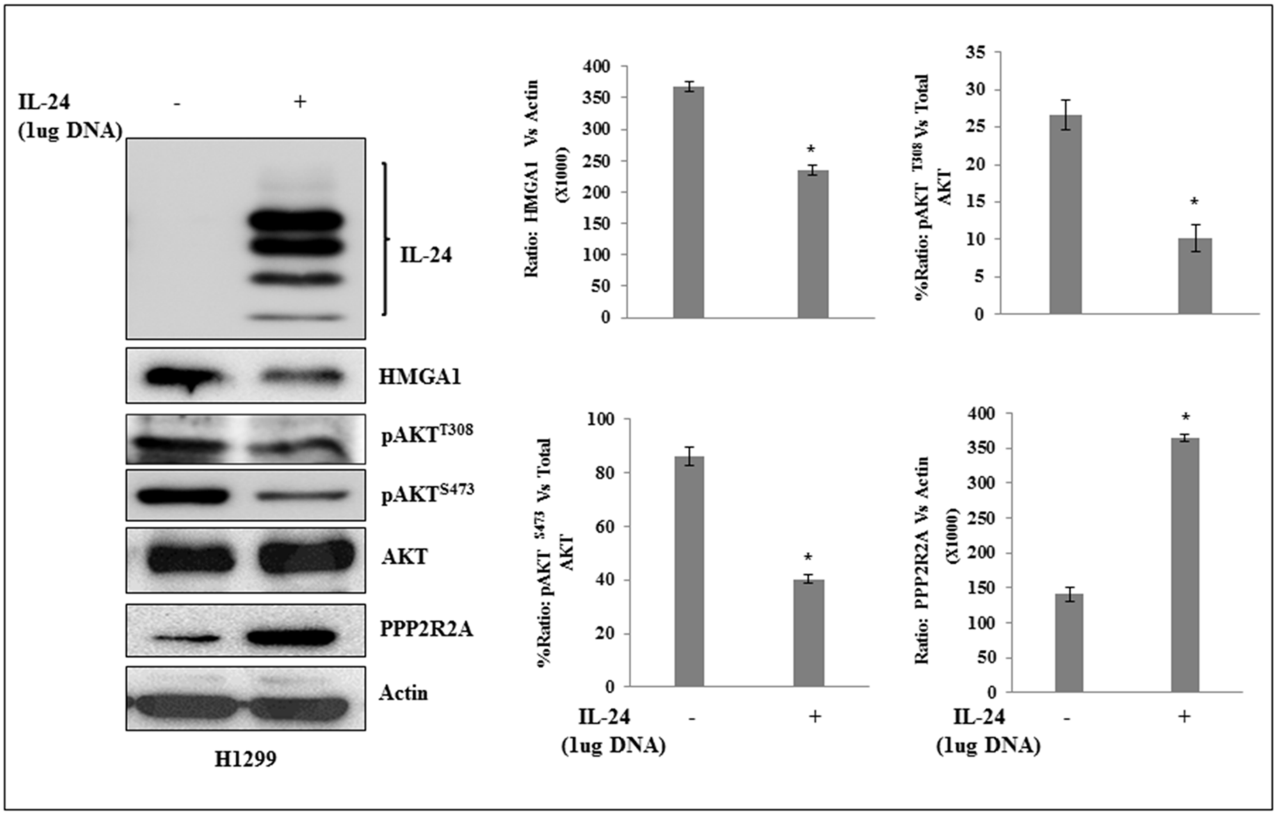

Supplementary Figure S2: Transient transfection of IL-24^{wt} plasmid inhibits HMGA1 and its downstream targets in H1299 cells. Transient transfection of IL-24^{wt} plasmid DNAreduced the expression of HMGA1, phosphorylated (p) AKT^{T308}, and pAKT^{S473}, and increased PPP2R2A expression in naïve H1299 cells compared with their respective non-transfected cells. Beta-actin was used as a protein loading control. Differences in the expression of the proteins were determined by semi-quantitative analysis and are presented in graphical format (p<0.05). Bars denote standard deviation (*SD*).

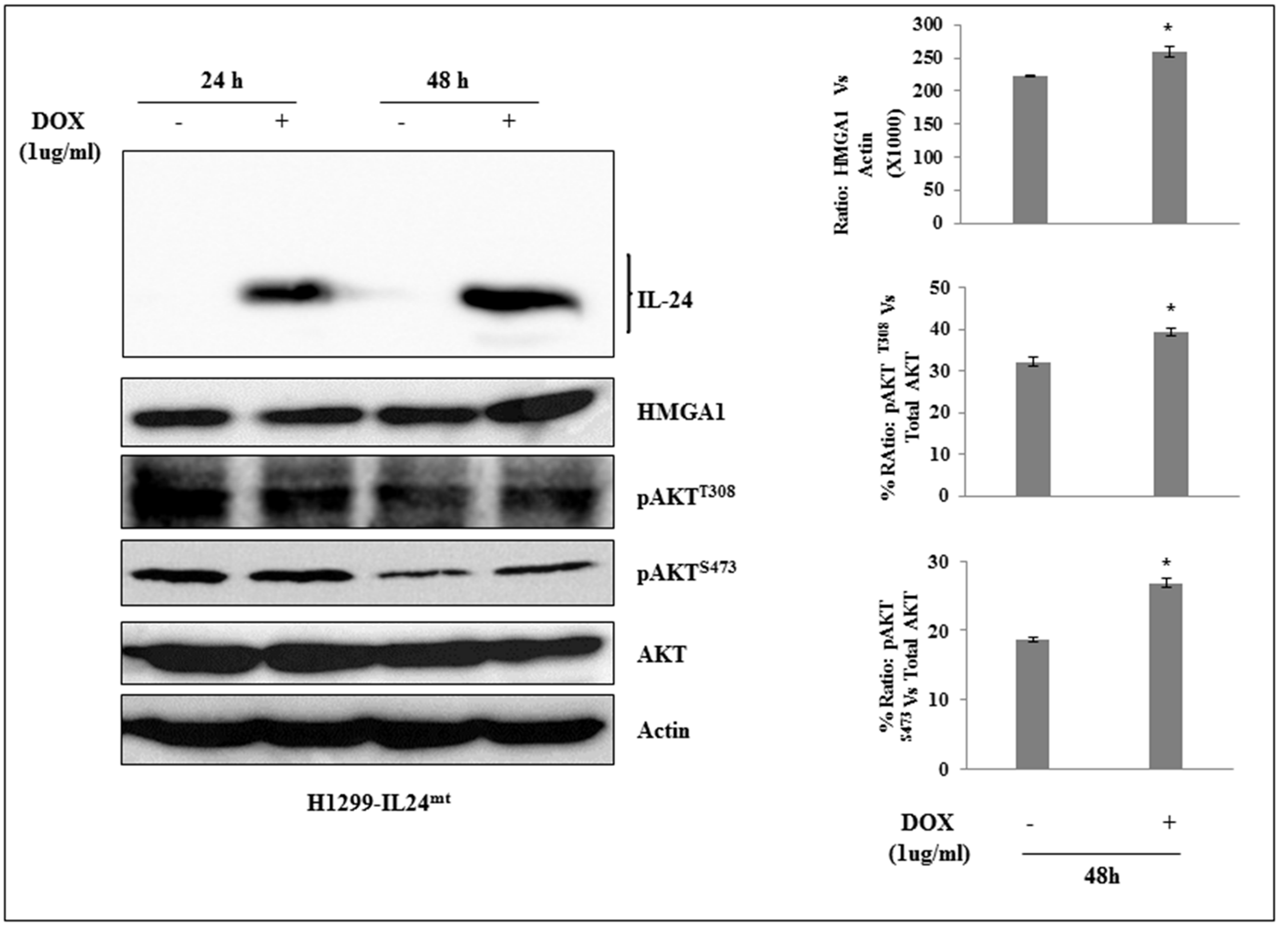

Supplementary Figure S3: IL-24 wild-type, but not mutant, suppressed HMGA1 expression in H1299-IL24^{wt} cells. Western blotting analysis showed that IL-24^{mt} protein expression in H1299-IL24 cells did not reduce the expression of HMGA1, phosphorylated (p) AKT^{T308}, or pAKT^{S473} at 24 h, but expression was markedly increased at 48 h after doxycycline treatment, compared with untreated control (p<0.01). Beta-actin was used as a protein loading control.

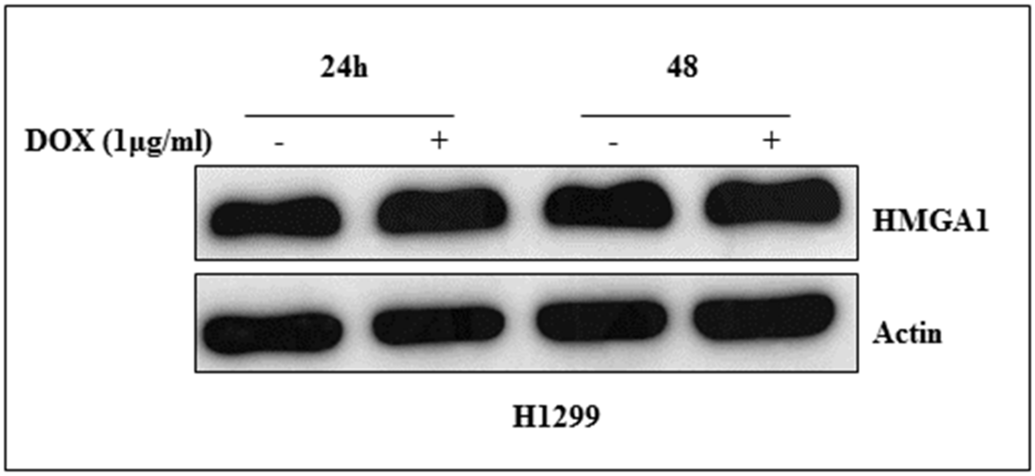

Supplementary Figure S4: Doxycycline alone does not inhibit HMGA1 expression in H1299 cells. The effect of doxycycline on HMGA1 expression was determined by treating naïve H1299 cells with 1 μg/ml doxycycline. Untreated cells served as controls. No significant HMGA1 inhibition was observed in doxycycline-treated cells compared with controls at 24 h or 48 h. Beta-actin was used as a protein loading control.

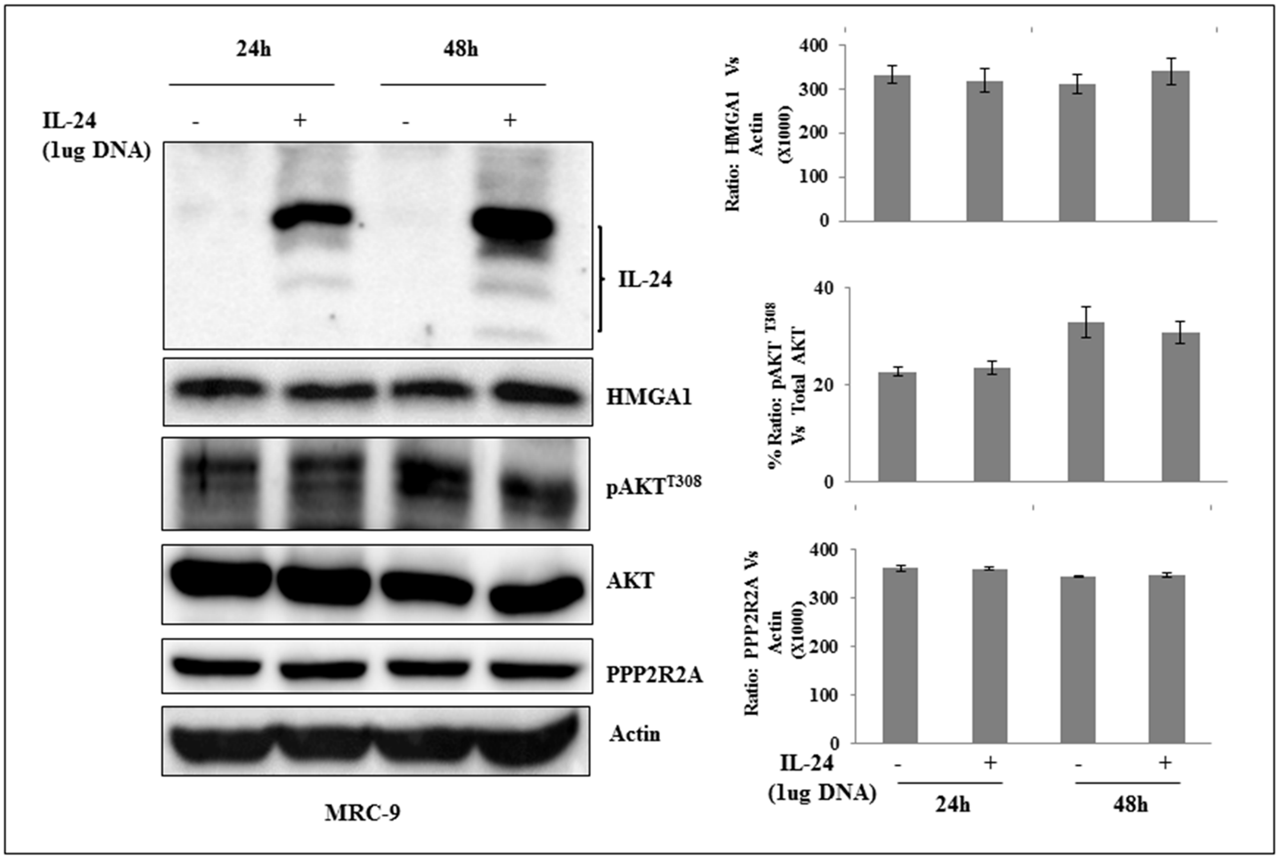

Supplementary Figure S5: Effect of IL-24^{wt} treatment on the HMGA1 axis in normal human lung fibroblast cells (MRC-9). Transient transfection of IL-24^{wt} plasmid DNA produced no noticeable change in the expression of HMGA1, pAKT^{T308}, or PPP2R2A in MRC-9 cells compared with their respective non-transfected cells. Beta-actin was used as a protein loading control.

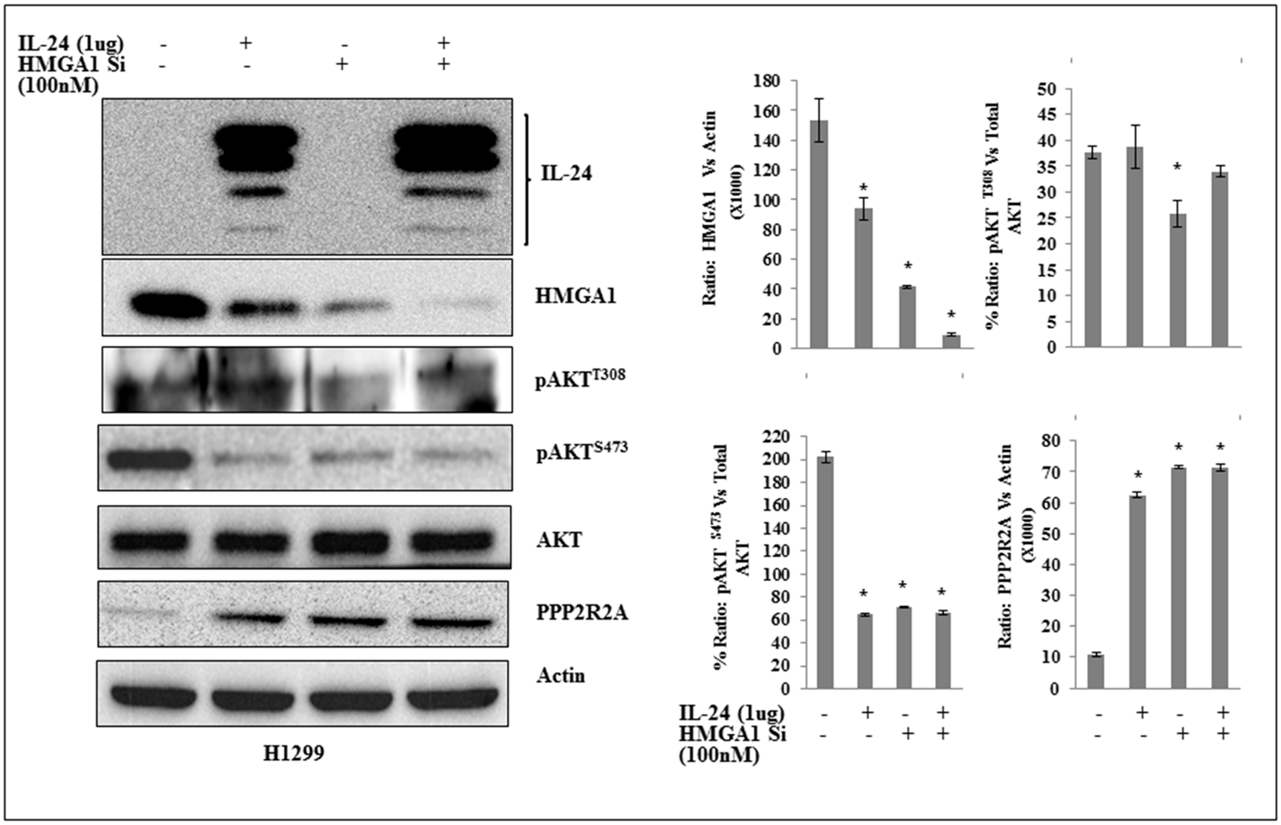

Supplementary Figure S6: A combination of IL-24 and HMGA1-siRNA showed greater inhibition of AKT activation in H1299 cells. Western blotting showed that all treatment groups showed reduction in the expression of HMGA1. Change in $pAKT^{T308}$, and $pAKT^{S473}$, and PPP2R2A expression was also observed in all three treatment groups compared with control. Beta-actin was used as a protein loading control. Differences in the expression of the proteins compared with untreated control were determined by semi-quantitative analysis and are presented in graphical format (p<0.05). Bars denote standard deviation (*SD*).

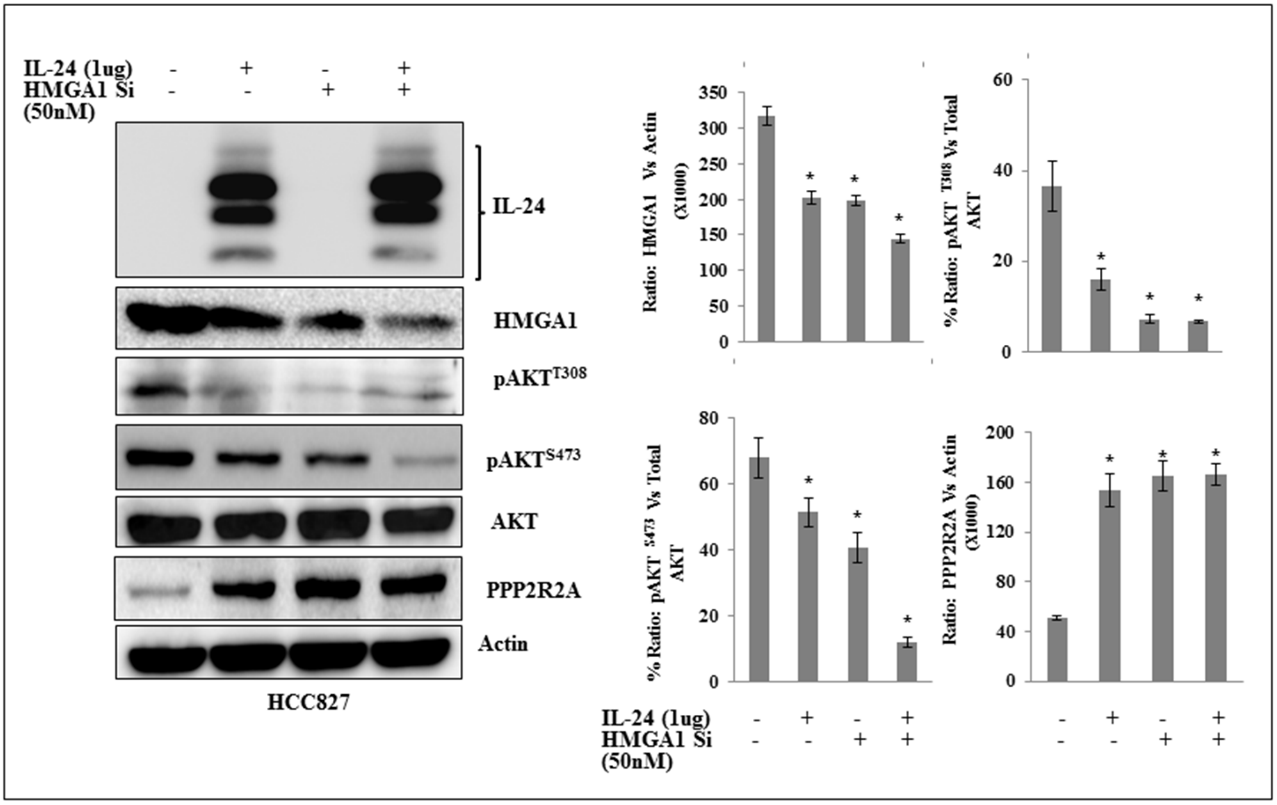

Supplementary Figure S7: The combination of IL-24 and HMGA1-siRNA showed greater inhibition of AKT activation in HCC827 cells. Western blotting showed that the combination of transient transfection of IL-24[wt] plasmid and HMGA1 siRNA produced greater reduction in the expression of HMGA1, and pAKT[S473] than was observed with all other treatments. Beta-actin was used as a protein loading control. Differences in the expression of the proteins compared with untreated control were determined by semi-quantitative analysis and are presented in graphical format (p<0.05). Bars denote standard deviation (*SD*).

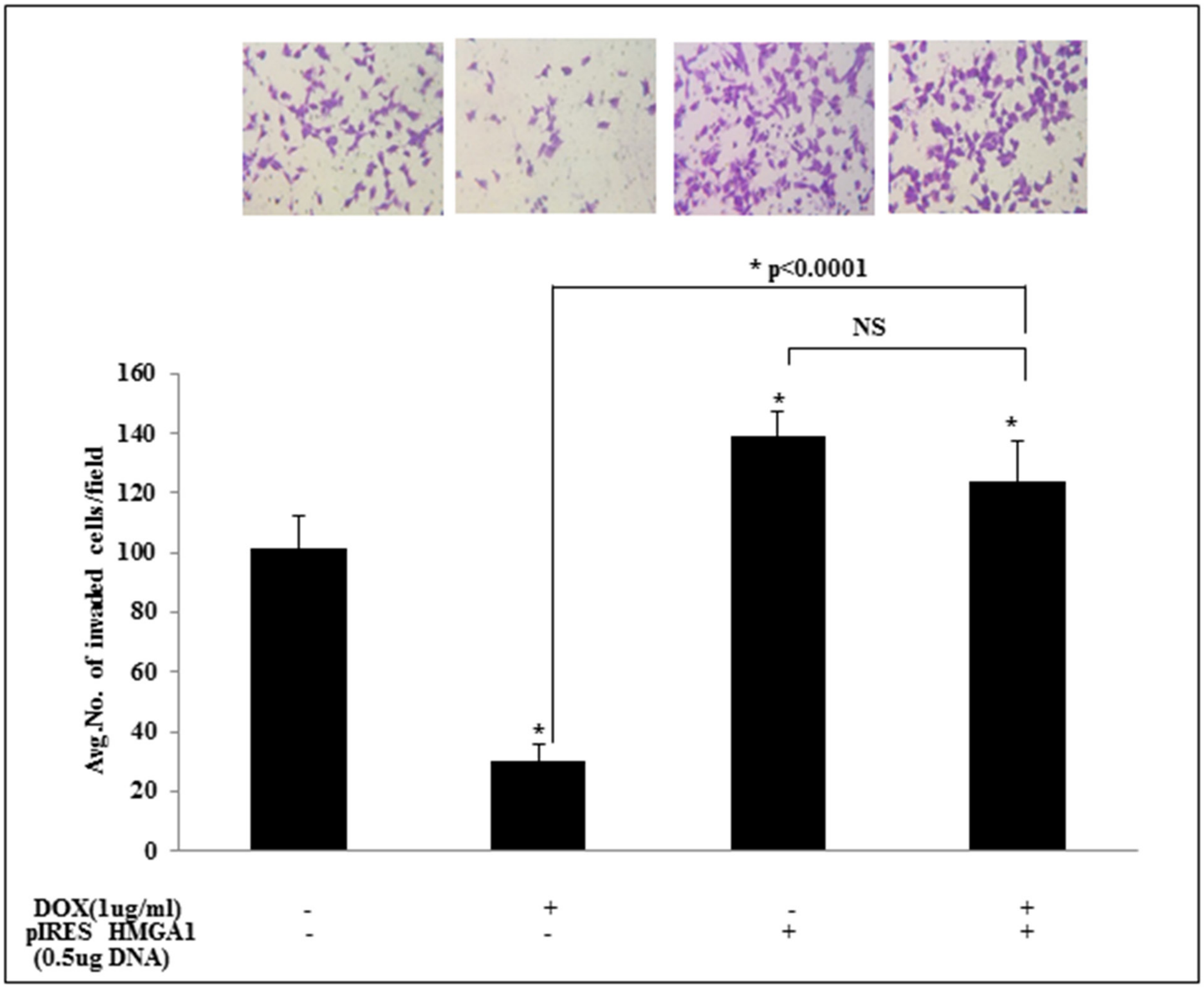

Supplementary Figure S8: HMGA1 overexpression rescues IL-24^{wt}-mediated inhibition on cell invasion. Overexpression of HMGA1 using the pIRES-HMGA1 expression plasmid in H1299-IL-24^{wt} cells resulted in significant abrogation of the IL-24^{wt}-mediated inhibitory activity on cell invasion ($p<0.05$). NS denotes "not significant".

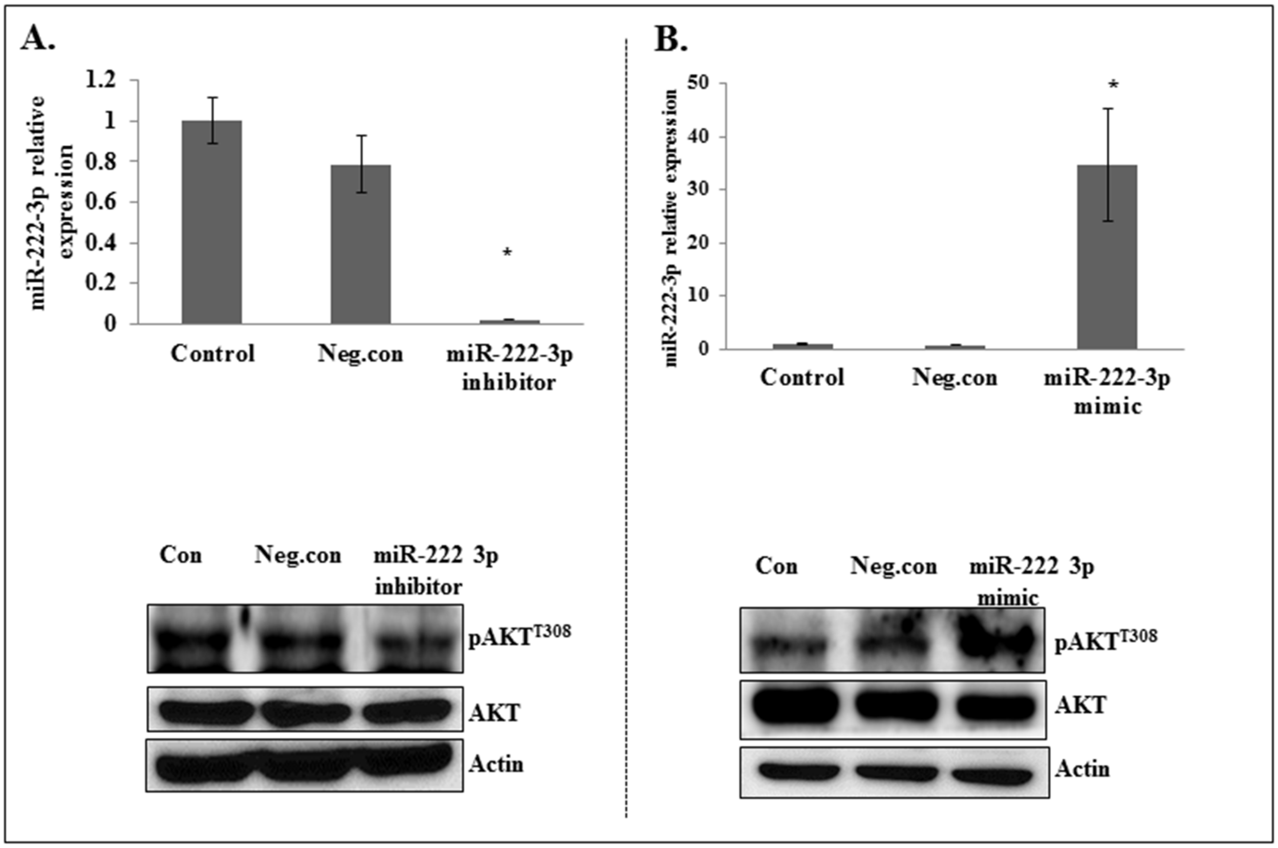

Supplementary Figure S9: miR-222-3p inhibitor and mimic are target-specific. A. RT-PCR and western blot analysis showed that the miR-222-3p inhibitor which was used to inhibit miR-222-3p expression in H1299- IL-24^{wt} cells was target-specific when compared with untreated and negative controls. Asterisk denotes significance (p<0.0001). **B.** RT-PCR and western blot analysis showed that the miR-222-3p mimic which was used to mimic miR-222-3p expression in H1299- IL-24^{wt} cells is target-specific in H1299-IL-24^{wt} cells, when compared with untreated and negative controls. Asterisk denotes significance (p<0.001). Beta-actin was used as a protein loading control (p<0.05).
